# Move the Neighbourhood: Study design of a community-based participatory public open space intervention in a Danish deprived neighbourhood to promote active living

**DOI:** 10.1186/s12889-017-4423-4

**Published:** 2017-05-19

**Authors:** Charlotte Skau Pawlowski, Laura Winge, Sidse Carroll, Tanja Schmidt, Anne Margrethe Wagner, Kamilla Pernille Johansen Nørtoft, Bettina Lamm, René Kural, Jasper Schipperijn, Jens Troelsen

**Affiliations:** 1 Research unit for Active Living, Department of Sports Science and Clinical Biomechanics, University of Southern Denmark, Campusvej 55, 5230 Odense M, Denmark; 2 Research Unit of Landscape Architecture and –Urbanism, Division of Landscape Architecture and Planning, Department of Geosciences and Natural Resource Management, University of Copenhagen, Rolighedsvej 23, 1958 Frederiksberg C, Denmark; 3 Institute of Architecture, Urbanism & Landscape, The Royal Danish Academy of Fine Arts, Schools of Architecture, Design, and Conservation, Philip de Langes Allé 10, 1435 Copenhagen K, Denmark

**Keywords:** Study design, Deprived neighbourhood, Active living, Urban installations, Co-design, Children, Seniors, SOPARC, Accelerometer, GPS

## Abstract

**Background:**

A limited amount of research has examined the effect of changing public open spaces on active living. This paper will present the study protocol of a community-based intervention study co-designed in an interdisciplinary collaboration with community members to develop urban installations highly tailored to promote active living among children (10–13-years-old) and seniors (>60-years-old) in a deprived neighbourhood in Copenhagen.

**Methods:**

The study builds on a quasi-experimental study design with two sub-studies: 1) a children study and 2) a senior study. The interventions will be developed, designed and implemented in collaboration with local children and seniors, respectively, using different co-design tools and methods. We will evaluate the effect of the interventions on children’s and senior’s use of the new-built urban installations using accelerometers in combination with GPS as well as systematic observation using the System for Observing Play and Recreation in Communities (SOPARC). A process evaluation with focus groups consisting of the various stakeholders in the two sub-studies will be used to gain knowledge of the intervention processes.

**Discussion:**

The paper presents new approaches in the field of public open space interventions through interdisciplinary collaboration, participatory co-design approach and combination of measurements. Using both effect and process evaluations the study will provide unique insights in the role and importance of the interdisciplinary collaboration, participatory processes, and tailoring changes in public open space to local needs and wishes. These results can be used to guide urban renewal projects in deprived neighbourhoods in the future.

**Trial registration:**

Retrospectively registered with study ID ISRCTN50036837. Date of registration: 16 December 2016.

## Background

Promotion of more active living in the last decade has become an important strategy to reduce the effect of increasingly sedentary lifestyles [1] that are responsible for more than 5 million deaths per year world-wide [2]. Active living is a way of life that reduces sedentary behavior (SB) and integrates more physical activity (PA) into daily routines. Active living includes things such as walking or cycling for transport; exercise or play for pleasure and fitness; sitting less during work or school hours; or sitting less at home [1]. Active living is associated with a multitude of positive short- and long-term health consequences due to its stimulating influence on physical, mental and social health [3–5].

How physically active people are in their daily life is related to many individual factors such as age, sex, socio-economic status (SES), but also on the characteristics of the physical environment they live in [6, 7]. It is documented that people with a low SES on average are less physically active compared to people with a high SES. This disparity in active living is aggravated when people live in neighbourhoods with low walkability [8]. The characteristics of public open space in the local neighbourhood are most important for those people that typically have the lowest mobility and are most sensitive to safety issues: children and elderly [9]. These two age groups are also relevant from a health perspective. Children’s low PA levels are worrying since PA patterns in early life are likely to track into adulthood [10] whereas older adults are the least physically active of any age group and generate the highest expenditures for medical care [11, 12].

The role of public open space in promoting active living has received increased attention in the past decade, but knowledge on how to improve public open space to increase active living is scarce and has been asked for [13, 14], in particular, to reduce social inequality in health [15, 16]. There are many reasons for this limited knowledge. First of all, developing and implementing changes in public open space is complex, expensive and takes time [17]. Second, creating changes that could have the desired effect requires involvement of many different participants (e.g., architects, planners, and public health professionals) traditionally not working together [14, 18]. Third, evaluating the effect of such changes requires an innovative study design and a wide range of methods [19, 20]. From a research perspective, we pose that neither of these challenges can be addressed satisfyingly by involving researchers from only one field.

To address these issues we developed the ‘Move the neighborhood’ study. The objective of this study is to collect research based knowledge on how to alter public open space in a deprived neighbourhood in Copenhagen to promote active living among people living there. Our target groups are children (10–13-years-old) and seniors (>60-years-old) living in the neighbourhood. In an interdisciplinary collaboration we developed a quasi-experimental intervention study that was inspired by the principles of Community-Based Participatory Research (CBPR) [21]. We use a co-design approach to develop highly tailored interventions in the form of urban installations [22].

### Aim

This paper will present the study protocol of the ‘Move the neighborhood’ study including a description of the case, co-design based development and implementation of urban installations, and measurements to be used in the evaluation of the study.

## Methods

### Research team

The study is conducted as part of the Activity- and health-enhancing Physical Environments Network (APEN), an interdisciplinary knowledge and development network. The partners in the network work at research units at three universities in Denmark: The Royal Danish Academy of Fine Arts, Schools of Architecture, Design, and Conservation - Institute of Architecture, Urbanism & Landscape; the University of Copenhagen - Division of Landscape Architecture and Planning; the and University of Southern Denmark - Research Unit of Active Living. The network consists of researchers with a broad range of backgrounds such as architects, landscape architects, designers, anthropologists and health researchers.

### Study design

**Fig. 1:**
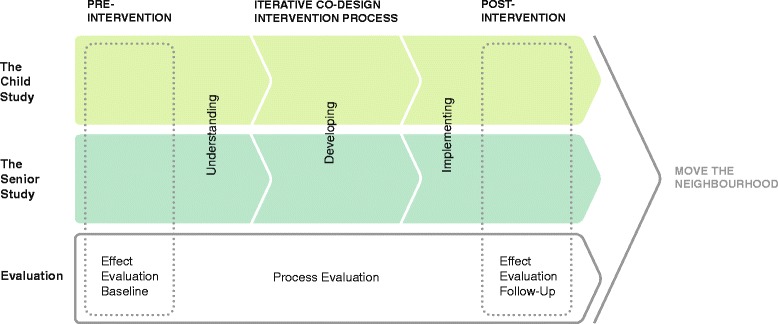
Illustration of study design

The study is a quasi-experimental community-based intervention study including two sub-studies: 1) a children study and 2) a senior study. Each of the two sub-studies is divided into three phases: 1) pre-intervention; 2) intervention; 3) post-intervention. The intervention phase includes different interlinked co-design steps: understanding, developing and implementing. Information collected during the first co-design step, understanding, will be used to start the second co-design step, developing. In this step researchers and participants will in an iterative process jointly explore and develop ideas where e.g. participant ideas will be visualised by designers and then presented back to participants to improve and decide upon. The ideas will be interpreted and implemented in the final step. The evaluation of the study is separated into an effect evaluation and a process evaluation. The effect evaluation will be conducted pre-intervention (baseline) and post-intervention (follow-up) to assess the effect of the interventions whereas the process evaluation will be conducted before, during and after the intervention phase to describe how the interventions were developed and implemented. To be able to support a comprehensive evaluation of the study, a range of qualitative and quantitative methods will be employed. The study design with its two sub-studies and phases is illustrated in Fig. 1.

### The neighbourhood

The intervention study takes place in the neighborhood of Sydhavn [the South Harbour] in Copenhagen, Denmark. The neighbourhood has 10,276 inhabitants [23], spans an area of 1.2 km^2^ and is framed by high-traffic corridors [24].

The district was planned and built between 1908 and 1950 in line with the welfare planning ideologies of that time in an effort to provide healthier and better living conditions for the growing work force moving into Copenhagen. With reference to the English garden cities the neighbourhood consists of homogenous 2–3 story brick buildings arranged in geometrical structures punctuated by green boulevards, small parks and public squares [23, 25]. Because of a decline in the number of inhabitants throughout the years the neighbourhood has become a place where socially challenged people are housed by public authorities [23]. Demographically the area is one of Copenhagen most disadvantaged neighborhoods. In this neighbourhood 22.1% of the population stands outside the labour market (17.1% on average in Copenhagen), 32.0% has no formal education (21.3% on average), and 40.2% has a low income (30.6% on average) [23]. With 73.0 years as average life expectancy, the neighbourhood has one of the lowest average life expectancies in Denmark where the average is 80.6 years [26].

As one of the last remaining deprived districts in Copenhagen that has not been subject to urban renewal, the area is selected to undergo large changes in the coming years through a municipal renewal initiative. Copenhagen Municipality will focus their investments in the neighbourhood on renewing urban green spaces, opening a Metro (subway) line in 2022, and a large park renewal project focusing on storm water management [23]. The public open space interventions planned in this study tie into this process, which allows for a close linkage between our intervention study and the municipal urban renewal strategies.

**Fig. 2:**
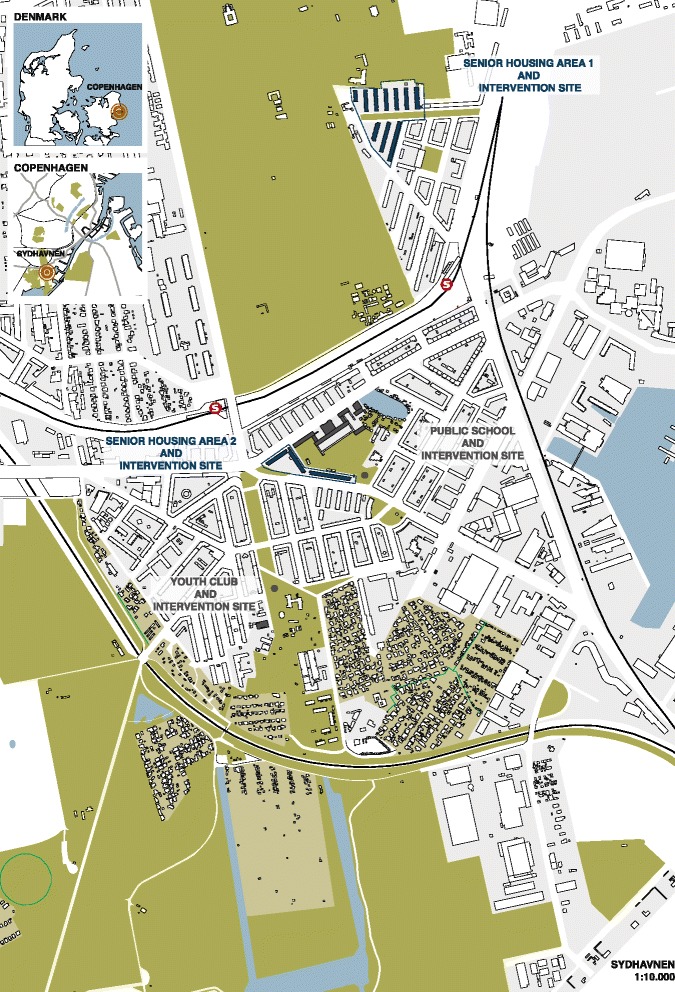
A map of the neighborhood

A map of the neighbourhood showing the different intervention sites is presented in Fig. 2.

### Iterative co-design intervention process

Throughout the three-step intervention phase we will use a community engaging approach called co-design that is increasingly used in urban development projects [27]. Co-design is a partnership approach that engages community members and designers in a joint design and implementation process [27]. In current study the community members are local children and seniors and the designers are researchers with professional backgrounds in design, architecture and landscape architecture who are part of the research team. The aim is to design and build urban installations in public open spaces that form new destinations in the neighbourhood that can support and increase movement, play, social interaction, quality of life and health for the community members through a process of designing *with* the users and not *for* them [27]. Co-design is a social process, with significant creative potential for innovation, which can be actively exploited when engaging community members in the design and construction process in public open spaces [28]. Various local key stakeholders, such as craft and design teachers, leader of the youth club, carpenters, members of a local government organisation called the Areal Renewal Office, social service managers and administrators at the two housing areas, have been identified and will be involved in study-related activities and meetings throughout the co-design intervention process. The stakeholders will be important in supporting the permitting of green space interventions and in the recruitment of participants [29]. The local stakeholders will be the link between the community members (children and seniors) and co-design researchers. The two sub-studies will be tailored to the two target groups, which will be elaborated on in the following.

#### Co-designing with children

In the children study, local children will be engaged throughout all intervention phases [30]. As part of the understanding phase, a pilot study was conducted in August 2016 at a local children’s culture festival to test and develop different co-design tools, such as mappings, creating collages, and building full-size prototypes in the public green space. The purpose of the pilot study was to gain insights into the children’s perceptions and experiences of their neighborhood [31, 32], to test co-design tools and to mobilise stakeholders by making the project visible in the community.

The development phase will contain workshops in two parallel processes where designers together with local children will use different co-design tools and methods from design practice to bring together insights and ideation in an iterative design process using mapping, collage-making, site explorations, sketching, and model making to design urban installations [33, 34]. One co-design process takes place at the local public school and includes two grade 5 classes (11–12-years-old, *n* = 39) during their craft and design classes, one day a week from January to May 2017. The second co-design process takes place in a local youth club among 10–13-years-old children (*n* = 20). The process will consist of workshops conducted in the youth club scheduled to take place once a week over a period of five weeks during spring 2017.

The implementation of interventions will also take place in collaboration with children, designers, and creators experienced in the execution of urban installations. Two locations are selected as intervention sites. The selection criteria were their immediate proximity to child oriented cultural public institutions (cultural house, school and youth club) and that they were embedded in accessible green spaces that already had spatial qualities that could serve as physical frames for the interventions (see the two sites on the map, Fig. 2). Approximately 110,000 USD is allocated to construct 2–3 urban installations targeting children.

#### Co-designing with seniors

In the senior study, local seniors will be engaged throughout all intervention stages. In the understanding phase lasting from September 2016 to March 2017 ethnographic fieldwork [35] in combination with individual go-along interviews using a GoPro camera in the neighbourhood (*n* = 16) [36–38] and a photo elicitation project (*n* = 20) [39] was carried out among seniors (>60 years old) living in two social housing areas. The participating seniors were recruited with help from the social staff working in each of the social housing areas. In order to gain knowledge with a wide perspective, we invited participants with an equal ratio of men/women and with different age-related impairments. These initial data serve a dual purpose by firstly making it possible to gain insights into the senior residents’ everyday life and e.g., identify perceived barriers and potentials for using their public open space, and secondly, by providing a visual dimension to the interviews that potentially can be part of the following co-design workshops [40–42]. Engaging the participants in the early stages of identifying barriers and potentials in their local neighbourhood will be the first step in preparing their mind-sets for the co-design process and will underline the importance of their input to the project. All participants will be invited to take part in the following design and development workshops along with other interested senior residents from the two social housing areas. This approach makes it possible to create a co-design process that provides a tailored perspective targeting the specific physical and social context.

The development process will consist of three workshops in each of the two social housing areas scheduled to take place every two to three weeks during a six to eight week period in April–May 2017. Different co-design tools and methods from design practice, such as mapping, making collages and building prototypes will be used to bring together insights and ideation in an iterative design process. Ideally, the co-design process will involve the same 20–30 seniors throughout the series of workshops, representing the two social housing areas in an equal ratio.

The open spaces within the two social housing areas have been selected as sites for the interventions (see the two sites on the map, Fig. 2). Given the potentially low physical abilities of the senior residents the implementation of the urban installations will be carried out by professionals, but the senior residents will be invited to participate in the construction based on their individual interests and capabilities. Approximately 110,000 USD is allocated to construct 2–3 urban installations targeting seniors.

### Evaluation measurements

As described above, the study consists of an effect evaluation and a process evaluation that each has their own data collection methods and measures, described in more detail below.

#### Effect evaluation

The aim of the effect evaluation is to examine the use of the urban installations developed and implemented with and for children and seniors. The System for Observing Play and Recreation in Communities (SOPARC) was used to systematically observe the use of selected locations in the pre-intervention phase (September–October 2016 and April–May 2017) and observations will take place in the post-intervention phase to systematically observe the use of the new urban installations and surrounding locations (September–October 2017 and April–May 2018). SOPARC is a validated tool to assess the use of public spaces in community settings [43]. SOPARC is used to record gender, age, PA level, primary activity and social interactions for each observed person. The compiled SOPARC data makes it possible to determine the number of users at different times and days of the week, as well as assess user characteristics, activity levels and common activities carried out by users at the observation locations. Furthermore, at the beginning of each observation, weather conditions were recorded and a picture was taken to visualise each area and its features. Observations take place at each of the four intervention sites (see Fig. 2) during three weekdays and one weekend day, four times a day (morning: 7.30–8.30, lunch: 11.30–12.30, afternoon: 15.30–16.30, and evening: 19.30–20.30). SOPARC data was collected over a five-week period by four trained observers and resulted in a total of 64 h of observations. SOPARC baseline data will be used to qualify the co-design workshops in the intervention process of the two sub-studies.

In the pre- and post intervention phase accelerometers and GPS are used to assess where participants spend their time and how physically active they are at those locations. For the children study, the ActiGraph accelerometer model GT3X+ is used to objectively measure PA with a 15 s epoch, and the QStarz BT-Q1000xt GPS device is used to record their location every 15 s. The GT3X+ is recognised as a valid and reliable tool for measuring children’s PA levels [44], and the Qstarz GPS device has shown good dynamic accuracy for recording locations [45]. The children are asked to wear the accelerometer and GPS in an adjustable elastic belt on their waist. For the senior study, two AXIVITY accelerometers model AX3 are used per person to objectively record PA and SB (raw data, 50 Hz). The AX3 device provides acceptable validity for measuring PA in seniors [46, 47]. The AX3 accelerometers are taped to the skin, one on the lower back and one on the thigh. We have chosen two skin taped AX3 devices for the senior study as they are more suitable than the ActiGraph used in the children study to identify SB (e.g. standing or sitting), which we expect will be the main behavior amongst seniors. The same Qstarz GPS device is used to measure the participating senior’s location every 15 s. The participating seniors are given several choices on how to wear the GPS device (on a belt, in an ankle holder, or on lanyard). All participating children and seniors are asked to wear the equipment for seven consecutive days, and only to take the GPS off when there is a risk of contact with water and at night when it is charging. To increase compliance the participants received short reminder text-messages on their mobile phones twice a day. For the baseline measurements 80 children and 37 seniors were recruited to wear accelerometers and GPS. The children were recruited through the local school. The seniors were recruited through local stakeholders who know the senior residents (i.e., social service managers at the two social housing areas) and by participating in their social activities in the neighbourhood.

During baseline data collection all participating children also completed an electronic questionnaire asking for background information and PA behavior during and after school hours. Participating seniors were interviewed in their homes, and the interviewers used an electronic questionnaire to ask about background information and barriers towards using their neighborhood. VERITAS, an interactive online map-based questionnaire, was used by the interviewers to identify daily mobility and social interactions [48].

#### Process evaluation

The aim of the process evaluation is to evaluate how the intervention has been implemented [49]. In the pre- and post-intervention phase focus group interviews will be conducted with participants that are part of the intervention process (local children and seniors, designers, creators), as well as local stakeholders (craft and design teachers, leader of the youth club, members of the Areal Renewal Office, social service managers and administrators at the two housing areas). Ideally the focus groups will include the same persons from each group (*n* = 4–8) before and after the intervention and will be conducted separately to give interviewees the possibility to speak freely. In total, four or five focus group interviews will be conducted before and after the intervention in each of the two sub-studies. The first focus group interview will focus on the different experienced challenges before the intervention, and the expectations for the coming development and implementation process. The second focus group interview will provide insights into the development and implementation process, experienced acceptability, appreciation and appropriation of the interventions. The focus groups will be conducted locally and will be audio-recoded. During the intervention phase participant observations will be conducted during workshops to gain an understanding of the context and intervention explored. This information will help guide the post-intervention focus group interviews [49].

### Data analysis

#### Effect evaluation analysis

SOPARC data will provide information on how specific places are been used and by whom, before and after the interventions, as well as the specific use of the implemented urban installations. For each observed area the total number of users will be compared, as will the share of children and seniors, the average activity level, and common activities.

The collected GPS and accelerometer data will be merged and processed using the Personal Activity and Location Measurement System (PALMS, https://ucsd-palms-project.wikispaces.com) to match the two types of data based on their timestamp, remove GPS error, classify accelerometer data into activity levels, and detect walking, cycling and vehicle trips. The combined accelerometer and GPS data will be further processed in a PostgreSQL database were GIS (geographic information system) data on all intervention sites, green spaces, and daily destinations will be incorporated as well. This will make it possible to assess which areas and destinations participants visited, how often they did this and how long they stayed. It will furthermore be possible to assess how active participants were during their visit to a location, and which mode of transportation they used to get there (see *Klinker* et al. 2014 for more information on this method) [50]. The differences between baseline and follow-up data will be analysed as repeated cross-sectional measures comparing the number of users, the share of children, the share of seniors, as well as average activity levels.

#### Process evaluation analysis

The pre- and post-intervention focus group data will be analyzed as a whole using a mix of text-based content analysis and thematic analysis [51]. Relevant themes connected to how the process was experienced by the various focus groups (participants and stakeholders in the intervention) will be extracted to identify barriers and facilitators during all stages from identifying the actual needs to designing and implement the urban installations.

## Discussion

The aim of this paper was to present the study protocol for the ‘Move the neighborhood study’, including a description of the case, the co-design based development and implementation of urban installations, and measurements to be used in the evaluation of the study. The study is a quasi-experimental intervention study in a Danish deprived neighbourhood in which a co-design approach will be used to develop highly tailored interventions in the form of urban installations. As there are many factors that can influence the results of this type of intervention, designing this study was complex and required us to make a multitude of decisions that will yield a series of benefits, but also will create some challenges that are further discussed below.

### The interdisciplinary collaboration

Creating changes in public open space that have an effect on active living requires involvement of many different participants [14, 18]. Other studies have shown that the more design and planning experiences are shared and have crossed professional barriers, the more likely the participants are to learn and generate valuable ideas, increase quality and flexibility, improve efficiency, and simulate appropriate use of resources [52]. A particular strength of the study presented in this paper is that it was planned and developed as an equal partnership between researchers from multiple fields, each using their core competences to share ideas and jointly complete all tasks in the study. The health researchers are responsible for the evaluation, and the designers, architects, landscape architects and anthropologists are responsible for the development and implementation of the intervention processes in collaboration with local children and seniors. From literature, the difficult point seems to be balancing the many different interests [52]. In our study we have already experienced that the differences between the scientific fields and traditions among the many different researchers involved in the study was challenging to bring together in a joint aim for the study. To solve this challenge we spent much time describing and explaining individual expectations and understanding each other’s views on research. One of the secondary goals of writing this protocol paper was that it forced us to discuss each step of the study and these open-minded dialogues have been beneficial in creating a common understanding of the study.

### Co-designing in deprived neighbourhoods

During the intervention phase principles of co-design will be used to develop and implement tailored interventions. Designing in a partnership between community members and designers has proven to be an effective approach for addressing social and cultural health inequalities in community-based interventions, particularly in deprived communities [53, 54]. Based on our previous experiences we knew that tailoring an intervention to local needs and wishes, and building on local engagement, were both crucial to the success of the intervention [55]. However, this participatory approach leads to a high degree of diversity in the intervention development process, which makes it difficult to plan the implementation and evaluation of the intervention. To be prepared for the many unknown issues that will arise conducting participatory research we tested co-design tools and collected background information about our research context and target groups in the understanding stage of the intervention phase. This information helped us to understand perceived barriers and potentials in the neighbourhood, which was advantageous for the following co-design workshops. For example, we learned that the children in particular were motivated to be involved in the creation of the installations and that the design workshops have to be very well prepared and clearly facilitated to maintain their attention. The seniors were difficult to involve for longer time periods and they had difficulties seeing themselves being part of the implementation phase. These experiences meant that the co-design process for development and implementation in the two sub-studies became more differentiated and more tailored to the specific target groups than originally intended. Our process evaluation will provide novel insights in the role and importance of the participatory co-design processes, tailoring changes to local needs and wishes.

### Learning from a single-case

The single-case study design is not suitable to provide reliable information that can be generalized to a broader population. However, we deliberately chose a single case to allow for a detailed examination where we can follow all intervention stages closely. In line with this, Flyvbjerg argued that even though knowledge cannot be generalized that does not mean that it cannot enter the collective process of knowledge accumulation in a given field [56]. Also in this field the use of case studies is becoming more common. For example, Sallis and colleagues used case studies to illustrate the potential for effective research translation to facilitate health-oriented land-use and transport practices and policies [7]. If our interventions are successful in achieving more active living in this neighbourhood we fully acknowledge that it will be difficult to implement this particular type of interventions on a larger scale. Nonetheless, the extensive evaluation of the use of specific installations will lead to new knowledge that can be used in urban renewal projects in deprived neighbourhoods in the future.

On a more critical note, Copenhagen Municipality will create many changes in the area in the coming years through an urban renewal process [23], so the current study is not the only project that will change public open space in the neighbourhood, which might make drawing conclusions more difficult. In the selection of our intervention location, we collaborated closely with the Areal Renewal Office so that these sites will not be the primary location for other changes to the urban environment in the neighbourhood. Furthermore, the timing and the selected effect evaluation methods, SOPARC observations and GPS in combination with accelerometer, make it possible to investigate changes in use of specific spots independent from the urban renewal projects.

### Measurements

Combining an effect evaluation and a process evaluation and collecting different types of data that complement each other is a strength of this study [17].

A novel aspect of our study is the combination of using SOPARC, accelerometer and GPS to determine our two target groups’ use of the new urban installations. Previously, a number of studies have used systematic observations to evaluate public open space interventions [57]. To our knowledge, using systematic SOPARC observations, accelerometer and GPS in combination has not been used before in longitudinal studies evaluating public open space interventions in neighbourhoods. Using accelerometer and GPS in combination has the potential to assess whether a change in active living is “relocated” activity (i.e. the same activity, but in a different location), or if it is a true increase in active living [55]. These methods also have the advantage that each individual is identifiable, which means that it is possible to adjust the analyses for different personal characteristics [50]. However, when using accelerometer and GPS no information about use of the newly built installations is obtained if our participants do not use the installations. Furthermore, the combination of accelerometer and GPS is relatively invasive for participants, which can complicate recruiting participants in the study and get sufficient valid data. Recruitment of children happened trough the local school and was relatively easy once the co-design collaboration had been put into place. However recruitment of seniors to wear the devices was highly challenging, despite our large efforts. Therefore, we supplemented our accelerometer and GPS data by collecting SOPARC data, which is much less invasive. SOPARC observations provide an overall picture of the use of specific areas and in the follow-up observations this method can provide information about the use of the specific urban installations that were built. Even though SOPARC is an objective observation tool, there might occur differences between different observers conducting the SOPARC observations. Especially age can be difficult to access by looking at a person for a few seconds. We have tried to compensate for that by having a workshop where the observers were trained in how to observe and assess age. After the training, all observers had to observe the same area at the same time and results were found to be comparable.

The information from the focus groups and the observation of workshops will be used to describe how the intervention has been implemented [49]. To get the most comparable baseline and follow-up viewpoints from the participants our intension is to include the same participants in our focus groups before and after intervention. However, in the understanding phase we found that it is very difficult to recruit and sustain seniors for longer time-periods, which may influence our recruitment of seniors for the follow-up focus group. For that reason, it is important to continue involving local stakeholders in study-related activities and meetings throughout the study to maintain the established links between researchers and community members [29].

## Conclusion

Evaluating a public open space intervention in a deprived neighbourhood is complex. This paper represents the ‘Move the Neighborhood’ study that has a new approach in this field by its interdisciplinary collaboration, participatory co-design approach and combination of measurements. The study will provide unique insights into the role and importance of the interdisciplinary collaboration, participatory processes, tailoring changes in active living to local needs and wishes. These results can be used to guide urban renewal projects in deprived neighbourhoods in the future.

## Abbreviations

CBPR: Community-Based Participatory Research
GIS: Geographic information system
GPS: Global Positioning System
PA: Physical activity
PALMS: Personal Activity and Location Measurement System
SB: Sedentary behavior
SES: Socio-economic status
SOPARC: System for Observing Play and Recreation in Communities
